# Improved outcomes in patients with chronic obstructive pulmonary disease treated with salmeterol compared with placebo/usual therapy: results of a meta-analysis

**DOI:** 10.1186/1465-9921-7-147

**Published:** 2006-12-29

**Authors:** Robert A Stockley, Philip J Whitehead, Michael K Williams

**Affiliations:** 1 Queen Elizabeth Hospital, Birmingham, UK; 2 GlaxoSmithKline, Greenford Road, Greenford, Middlesex UB6 OHE, UK

## Abstract

**Background:**

Several studies have demonstrated that long-acting β_2_-agonists such as salmeterol are beneficial in chronic obstructive pulmonary disease (COPD). A meta-analysis was therefore conducted to review studies in COPD to provide pooled estimates of the effect of salmeterol 50 mcg taken twice daily in addition to usual therapy on several clinically relevant endpoints, when compared with placebo/usual therapy.

**Methods:**

An extensive search of literature and clinical trial databases was conducted using the terms salmeterol, COPD, chronic, obstructive, bronchitis and emphysema. Nine randomized, double-blind, parallel-group, placebo-controlled trials of ≥12 week duration with salmeterol 50 mcg bid treatment in COPD were included (>3500 patients), with a further 14 trials excluded due to study design or reporting timelines. All patients were included, and a sub-group of subjects (84%) with poorly reversible COPD were considered separately. Statistical testing was carried out at the 5% level, except for interaction testing which was carried out at the 10% level.

**Results:**

Patients treated with salmeterol over 12 months were less likely to withdraw early from the studies (19% patients compared with 25% on their current usual therapy, p < 0.001), less likely to suffer a moderate/severe exacerbation (34% compared with 39%, p < 0.0001) and had a greater increase in average FEV_1 _(73 mL difference vs placebo/usual therapy, p < 0.0001). Similar differences were found at 3 and 6 months. At all time points, more patients experienced an improvement in health status and also a greater change with salmeterol than with placebo/usual therapy (p < 0.002). There was no evidence of tachyphylaxis to salmeterol over 12 months.

**Conclusion:**

The meta-analysis confirmed clinically and statistically significant, sustained and consistent superiority of salmeterol 50 mcg bid over placebo/usual therapy on a broad range of outcome measures.

## Background

Chronic obstructive pulmonary disease (COPD) is a debilitating progressive multi-component disease characterised by airflow limitation that is not fully reversible [1]. COPD is associated with an inflammatory response in the airways, together with airway obstruction, mucociliary dysfunction and structural changes within the lungs [1]. These features result in combinations of symptoms and physiological changes that affect the ability of patients to function, and ultimately influence survival. In addition to chronic symptoms, patients with COPD may experience acute exacerbations, which have a major impact on morbidity, mortality and healthcare utilization [2-4].

The burden of COPD is considerable. Currently, COPD is the fourth leading cause of mortality worldwide, predicted to rise to the third leading cause by 2020 [5]. As a disabling condition that affects the physical and social functioning of the sufferer, COPD is also associated with considerable impact on health status. Thus by 2020, COPD is projected to be the fifth leading cause of disability [5].

With increasing understanding of the pathophysiology of COPD, a number of pharmacological and surgical approaches to management of the disease have been developed. International management guidelines recommend that the goals of treatment should be to prevent and control symptoms, prevent and reduce the severity of acute exacerbations, improve lung function, and improve health status [1]. In the design of clinical trials, endpoints that are considered to be clinically important have also been clarified recently, and include withdrawal rates from studies, exacerbation rates, lung function (pre and post-bronchodilator FEV_1_) and health status [6].

More recent therapies for COPD include salmeterol, a long-acting inhaled β_2_-agonist, anticholinergic agonists such as tiotropium bromide and others such as Phosphodiesterase 4 inhibitors which have resulted in a large body of published data assessing the efficacy from numerous clinical trials. These trials have varied considerably in terms of inclusion and exclusion criteria, endpoints and study duration and often restrict other therapies in order to demonstrate any advantage of the new therapy which provides an impression of efficacy that may not reflect the "real world". However, there have been several controlled trials of salmeterol therapy added to usual treatment in COPD which more likely reflects usual prescribing practice. The opportunity was therefore taken to review these studies in the light of current knowledge about relevant clinical endpoints.

The aim of the subsequent meta-analysis of patients with COPD was to provide pooled estimates of the effect of salmeterol 50 mcg taken twice daily when compared with placebo/usual therapy on several clinically relevant endpoints.

## Methods

### Data sources

An extensive literature search was conducted through the database, Medline, using the terms salmeterol, COPD, chronic, obstructive, bronchitis and emphysema, with the full reference (where available) used to review the study results. The same terms were also used to search the GlaxoSmithKline clinical trial tracking system. All completed studies reported by 7 January 2002 were included in the analysis.

### Study selection

Studies were included in the analysis if they met the following inclusion criteria:

1) Randomized, double-blind, parallel-group, placebo-controlled trial of at least 12 weeks duration

2) Data was available on at least one of the specified endpoints (withdrawal rate, moderate/severe exacerbations, pre-bronchodilator FEV_1 _and health status)

3) Patients were non-asthmatic adults with stable COPD and no recent infections, exacerbations or hospitalisations in the previous 4 weeks (studies including subjects with other severe conditions, including cardiac, liver and renal disease were also excluded)

4) At least two treatment arms: salmeterol 50 mcg bid and placebo (with or without usual therapy).

**Figure 1:**
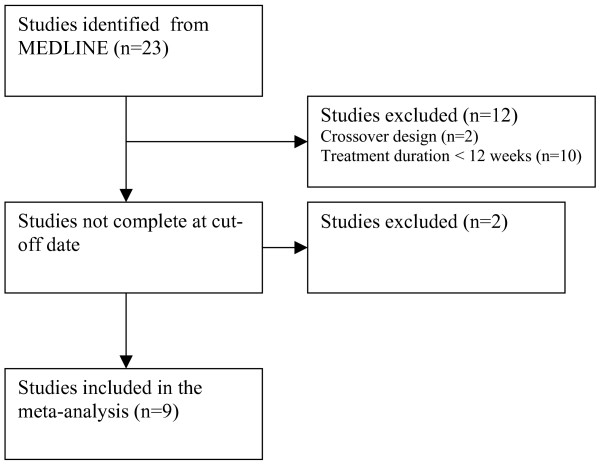
Flow diagram of the trial selection process.

Based on these criteria, nine studies were identified for inclusion in the meta-analysis [7-16], with 14 studies identified but not included in the analysis due to their crossover design, short treatment duration, or incomplete status at the cut-off date (Figure 1). All the studies that met the criteria had been sponsored by GlaxoSmithKline.

### Data extraction

Analysis of individual subject data was performed in all cases. Data relating to two populations were extracted, corresponding to the American Thoracic Society [17] and European Respiratory Society [18] definitions of COPD, respectively:

• Intention to treat (ITT) population, which consisted of all randomized subjects taking ≥ 1 dose of study medication

• Poorly reversible (PR) population, which was defined as the sub-group of subjects with reversibility <10% of predicted FEV_1 _following inhalation of albuterol 400 mcg or equivalent.

Information on a number of covariates was also extracted to facilitate subgroup analysis, including age of subjects, baseline FEV_1_, body mass index, duration of COPD, smoking history and use of regular inhaled and oral corticosteroids at baseline.

Analysis of data on FEV_1 _and health status included the actual data recorded at each time point (Observed), and, to account for withdrawals, the last observation carried forward to each time point (LOCF). LOCF was the primary analysis and is presented here. There were no meaningful differences between LOCF and Observed analyses.

Analyses were conducted for treatment of 3, 6 and 12 months duration. The visit identifier on the study database was used to allocate each observation to a time point. In the study reported by Stockley et al. [15,16], data for the 3- and 6-month endpoints were calculated by linear interpolation of data from the 1- and 4-month assessments, and 4- and 7-month assessments, respectively, as 3- or 6-month assessments were not performed.

The recording of exacerbations data differed from study to study. To make the data comparable, case record forms were examined and consistent definitions applied (**mild**: managed by the subject by modifying the dose of COPD medication; **moderate**: required the use of additional oral corticosteroids and/or antibiotics; **severe**: required hospitalisation) [19]. For the FEV_1 _and reversibility data, the mean pre-treatment values were used as the baseline. In the European studies [10,13-16], the St George's Respiratory Questionnaire (SGRQ) was used to assess health status, whereas studies conducted in the USA [8,9,11,12] used the Chronic Respiratory Disease Questionnaire (CRDQ). To combine data from these studies, the measure used was the proportion of subjects achieving a clinically significant improvement in health status (4-point or more decrease from baseline in the total score for the SGRQ; 10-point or greater increase in total score for the CRDQ) [20-23]. In addition, the change from baseline has been expressed as a percentage of the clinically relevant difference for each measure in order to combine the information from both types of questionnaire. The study reported by van Noord [7] did not include a health status assessment and was thus excluded from the analysis of this endpoint.

### Data analysis

All analyses were performed, using SAS 8.1 on a Unix environment. The primary model was fixed effects, with testing for interaction between treatment and study indicator to see if this was appropriate for each analysis. A random effects model was fitted if there was heterogeneity. Statistical testing was carried out at the 5% level, except for interaction testing which was carried out at the 10% level.

The analysis examined only moderate and severe exacerbations, due to their greater clinical significance and objectivity (assessment by physician required). Proportional hazards modelling was used to estimate the difference in time to first exacerbation for individual studies and for all studies, and calculate the relative risk between treatments. This approach was also used to analyse time to withdrawal from studies. For health status, a repeated measure analysis was carried out. Summary statistics are presented as means or percentages, as relevant, including standard deviations (SD).

### Results

#### Study characteristics

Table 1 summarises the nine studies included in the meta-analysis, together with the main eligibility criteria. The study reported by Boyd [10] included a criterion for reversibility to albuterol (5–15% change in baseline FEV_1_), as did studies reported by Calverley and Stockley [14-16] (≤ 10% change in predicted FEV_1_). The study reported by Stockley [15,16] also included a requirement for two or more moderate or severe exacerbations in the previous year, while the study reported by Calverley [14] required at least one exacerbation per year for the previous 3 years. Studies reported by Mahler, Hanania, Calverley and Boyd [10-12,14] included a requirement for cough and phlegm for 3 or more months a year during the previous 2 years, while studies reported by Chapman and Stockley [13,15,16] specified sputum for 3 or more months a year in the previous 2 or more years. All studies specified a smoking history of at least 10 pack years, with studies reported by Mahler and Hanania [11,12] requiring a history of at least 20 pack years.

**Table 1:** Studies included in the analysis

Study	Duration (weeks)	No. in ITT (PR) populations		Main eligibility criteria				COPD medication allowed during the study			
		
		Salmeterol	Placebo	Definition	Age (yrs)	FEV_1 _(% predicted % absolute value)	FEV_1_/FVC	OCS	ICS	Antichol.	MX
van Noord	12	49 (40)	50 (44)	ATS	40–75	≤65% & ≥ 0.75L	≤60%	Yes	≤ 1 mg/day FP*	No	Yes
Rennard	12	131 (84)	133 (85)	ATS	≥35	≤ 65% & > 0.7L or ≥ 40% & <0.7L	≤70%	≤ 10 mg prednisolone*	Yes	No	No
Mahler	12	135 (78)	143 (90)	ATS	≥35	≤65% & >0.7L or ≥ 40% & <0.7L	≤70%	≤10 mg prednisolone*	Yes	No	No
Boyd	16	228 (221)	227 (215)	ERS	40–75	≤70% & >0.6L	≤60%	Yes	Yes	Yes	Yes
Mahler	24	164 (105)	185 (130)	ATS	≥40	<65% & >0.7L or >40% & ≤0.7L	≤70%	No	No	No	Yes
Hanania	24	176 (112)	185 (118)	ATS	≥40	<65% & > 0.7L or >40% & ≤0.7L	≤70%	No	No	No	Yes
Chapman	24	201 (173)	206 (171)	ERS	≥40	≤85%	≤70%	Yes	Yes	Yes	Yes
Calverley	52	372 (371)	361 (359)	ERS	40–79	≥25%–≤70%	≤70%	Exacerbations	No	Yes	Yes
Stockley	52	316 (292)	318 (304)	ERS	≥40	<70%	Not stated	Exacerbations	≤1 mg/day FP*	Yes	Yes
Pooled		1772 (1476)	1808 (1516)								

* or equivalent; ATS: American Thoracic Society; ERS: European Respiratory Society; FEV_1_, forced expiratory volume in one second; FVC, forced vital capacity; OCS: oral corticosteroids; ICS: inhaled corticosteroids; Antichol: anticholinergics; MX: methylxanthines. FP: fluticasone propionate

In addition to the study medication and placebo, usual COPD therapy was continued in most of the trials, generally with the requirement that doses were kept stable. Short-acting bronchodilators were allowed in all trials, but not as a combination with other drugs except in the study reported by Chapman [13]. Antibiotics for acute exacerbations were permitted in all trials. Regular treatment with oral and/or inhaled corticosteroids was permitted in six of the trials (67%), while anticholinergic therapy could be continued in four trials (44%) and methylxanthines in seven trials (78%). Consequently, the analysis is effectively a comparison of adding salmeterol or placebo to usual therapy and so more closely approximates the situation in normal clinical practice.

Salmeterol and placebo were administered via Chlorofluorocarbon containing pressurised metered dose inhaler in four studies [7-10] whereas the Diskus™/Accuhaler™ dry powder inhaler was used in all other studies [11-16]. Both devices are licensed to deliver the same dose of salmeterol (50 mcg bid) in COPD.

A total of 3580 patients were included in the analysis, with 2565 males (72%). Demographic and baseline characteristics were well-matched between treatment groups, with an average age of 63.8 years (SD ± 8.8), baseline FEV_1 _of 1.29L (± 0.47) which was 45.2% predicted (± 13.8) and an average baseline reversibility of 5.96% predicted (± 5.2%). Most subjects (2474; 69%) had a diagnosis of COPD of 5 or more years duration, 46% were using inhaled corticosteroids prior to study start and 4% were using oral corticosteroids. Mean smoking history was 49.1 pack years (± 29.6). The characteristics of the PR population (84% of total) were not significantly different to the ITT population.

### Withdrawal from studies

**Figure 2:**
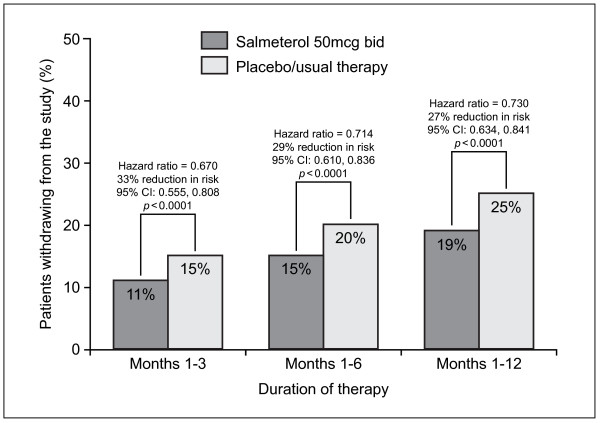
Cumulative withdrawal from clinical studies (pooled results from Intent To Treat population).

For all three time periods studied (1–3, 1–6 and 1–12 months), there was a consistent and highly statistically significant reduction in the percentage of early withdrawals in the ITT population treated with salmeterol compared to usual therapy, as summarised in Figure 2 (p < 0.0001 at all time points). Results were similar with the PR population. In both populations (ITT and PR), fewer patients on salmeterol withdrew over months 1–6 if they had a higher body mass index (BMI). In the ITT population, the risk of withdrawal following treatment with salmeterol was reduced by 35% in patients with BMI>27 (p = 0.0017) and by 55% in patients with BMI>24-<27 (p < 0.0004), but only 10% for patients with BMI<24 (p = 0.3971).

### Exacerbations

**Figure 3:**
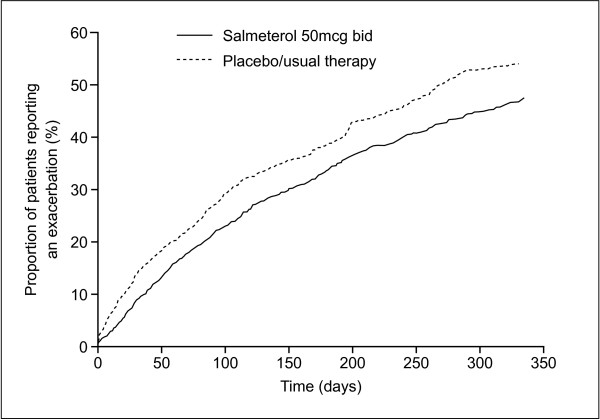
Survival analysis of time to first exacerbation (pooled results from Intent To Treat population).

Survival analysis showed that use of salmeterol delayed the time to first exacerbation (Figure 3). At each time point analysed, there was a consistent and highly significant reduced risk of moderate or severe exacerbation in the ITT population treated with salmeterol compared with usual therapy (28% during months 1–3, 24% during months 1–6 and 22% during months 1–12, p < 0.0001) Results were similar with the PR population (20–25% reduction in risk compared with usual therapy, p = 0.0002). All types of subjects showed similar level of reduced exacerbation risk irrespective of their disease severity, smoking history, duration of COPD or current therapy.

### Lung function

**Figure 4:**
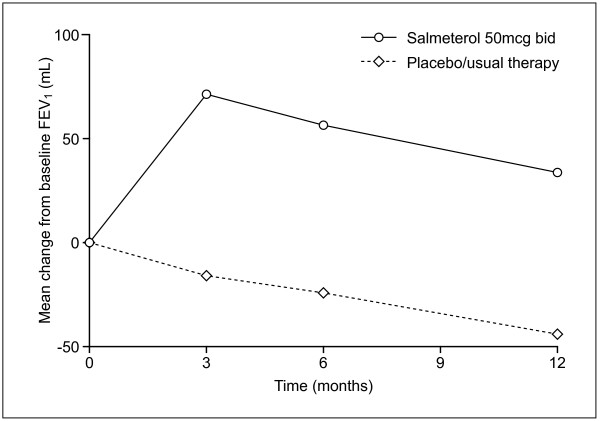
Mean change from baseline FEV_1 _(pooled results from Intent To Treat population).

**Figure 5:**
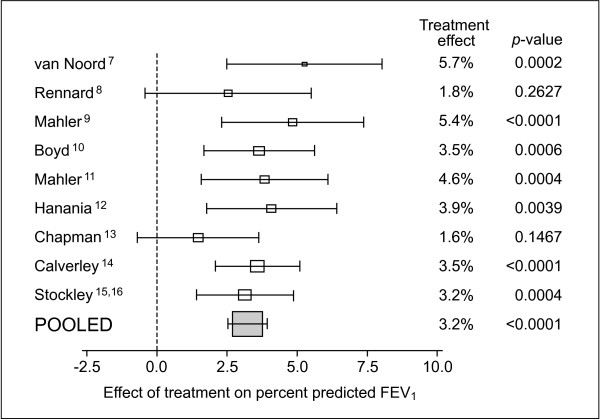
Percent predicted FEV_1 _(Intent To Treat population).

There was a consistent and statistically significant increase in average pre-bronchodilator FEV_1 _with salmeterol treatment compared with usual therapy, ranging from 73–86 mL over the three time periods assessed (Table 2, Figure 4). This corresponded to increases of 3.2% (Figure 5), 3.0% and 3.0% as a percentage of predicted FEV_1 _after 3, 6 and 12 months, respectively (p < 0.0001). Results were comparable in the PR population (increase in pre-bronchodilator FEV_1 _of 68–74 mL or 2.9–3% percent predicted). The youngest subjects (<60 years; n = 1001) had a consistently greater difference in favour of salmeterol (range 111–113 mL, p < 0.001) than the oldest (>70 years, range 43–59 mL, p = 0.0083; n = 826), with intermediate values for the subjects aged 60–69 years (n = 1316) Previous (not concurrent) use of inhaled corticosteroids was associated with smaller treatment differences at most time points, possibly due to the greater severity of COPD in subjects when ICS are more likely to be prescribed. The average pre-bronchodilator FEV_1 _for patients on placebo/usual therapy showed a decrease below baseline at all time points (Figure 4).

**Table 2:** Mean difference in pre-bronchodilator FEV_1 _between treatment with salmeterol and with placebo/usual therapy after 3, 6 and 12 months of treatment (intent to treat population).

Study	No. of subjects		Mean treatment effect on pre-bronchodilator FEV_1 _(ml)					
	
	Salmeterol	Placebo	3 months	P value	6 months	P value	12 months	P value
van Noord	49	50	108	0.0056	-	-	-	-
Rennard	131	133	74	0.0027	-	-	-	-
Mahler	135	143	128	<0.0001	-	-	-	-
Boyd	228	227	102	<0.0001	-	-	-	-
Mahler	164	185	96	<0.0001	94	<0.0001	-	-
Hanania	176	185	97	<0.0001	92	<0.0001	-	-
Chapman	201	206	42	0.0278	20	0.3559	-	-
Calverley	372	361	80	<0.0001	91	<0.0001	61	0.0022
Stockley	316	318	81	<0.0001	74	0.0002	96	<0.0001
Pooled	1772	1808	86	<0.0001	75	<0.0001	73	<0.0001

-: not applicable

### Health status

**Figure 6:**
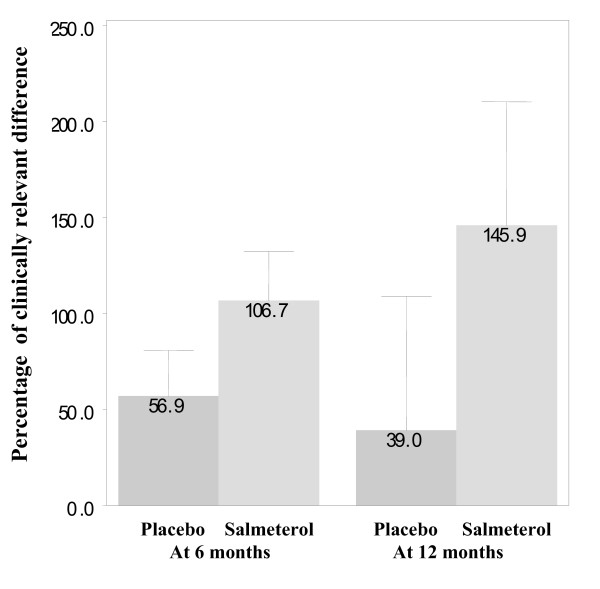
Percentage of clinically relevant difference in health status (change from baseline).

There was a consistent, statistically significant and clinically meaningful improvement in health status with salmeterol compared with usual therapy (Table 3). Health status improved beyond the clinically significant thresholds with salmeterol therapy in about half of the ITT population (46%) compared with 38% experiencing such an improvement with usual therapy (p < 0.0001). Similar results were found in the PR population (45% compared with 39%, p < 0.0016). Among the ITT population, the greatest benefits with salmeterol were noted in younger subjects (11% difference in those aged <60 years, p = 0.007 vs 3–4% difference for those aged >70 years, p = 0.3649) and those with greatest reversibility (8% difference for subjects with >5% reversibility, p = 0.0031 vs 5% for those with <5% reversibility, p = 0.1587). Among those patients completing the SGRQ, the percentage of subjects with a meaningful improvement over 12 months also favoured salmeterol (ITT 46.5% vs 38.5%, p = 0.0118; PR subjects 46.1% vs 38.9%, p = 0.024). In addition, the change from baseline expressed as a percentage of the clinically relevant difference for each measure (4 point decrease for SGRQ and 10 point increase in CRDQ) at 6 and 12 months is shown in Figure 6 indicating not only that increased numbers of subjects on salmeterol passed the clinically meaningful threshold but that the average increase was also greater.

**Table 3:** Proportion of patients experiencing a clinically meaningful change in health status with salmeterol or placebo/usual therapy.

Time period	Salmeterol		Placebo		Pooled estimate of difference		
	
	No. of subjects	% with meaningful change	No. of subjects	% with meaningful change	Difference in % meaningful change	95% CI	P value
Intent to treat population							
Weeks 8–28	1150	45.3%	1129	37.9%	7.7%	4.6, 10.7	<0.0001
Weeks 8–52	1156	45.9%	1131	37.9%	7.9%	5.1, 10.7	<0.0001
Poorly reversible population							
Weeks 8–28	934	44.6%	935	38.9%	5.4%	2.1, 8.8	0.0016
Weeks 8–52	939	45.3%	937	39.0%	5.9%	2.8, 8.9	0.0002

## Discussion

Evidence from a meta-analysis of randomized, controlled clinical trials is usually considered the most influential in management guidelines because of the large number of patients involved. This meta-analysis included 3580 patients recruited from centres in 34 countries across four continents, and showed consistent and statistically significant superiority of salmeterol 50 mcg bid over placebo/usual therapy on all outcome measures evaluated after 3, 6 and 12 months of treatment. Patients were 27–33% less likely to withdraw early from a study (p < 0.0001) and 22–28% less likely to suffer a moderate or severe exacerbation (p < 0.0001) when salmeterol rather than placebo was added to usual therapy. Lung function improved by 73–86 mL (3.0–3.2% predicted FEV_1_) and more patients experienced a clinically meaningful improvement in health status when treated with salmeterol compared with placebo/usual therapy (46% vs 38%, p < 0.0001).

The results of this meta-analysis are likely to be widely applicable to patients with COPD. Each of the nine studies included was sufficiently powered to detect a pre-defined difference in one or more of the efficacy measures of interest. Heterogeneity in the results from different trials was observed only for FEV_1 _during months 1–6, though the degree of departure was small and the results derived from the random effects model did not differ from those of the fixed effects model.

Demographic and baseline characteristics were well-matched between treatment groups, with the study population reflecting the profile of typical COPD patients. Over half of the subjects had been randomized into studies with duration of 24 weeks or more, and 38% were recruited into studies of 1-year duration. Consequently, a reasonable assessment of long-term (6–12 months) efficacy of salmeterol can also be derived from the results of this meta-analysis. The ITT population corresponded to subjects meeting the American Thoracic Society definition of COPD [17], while the subset of subjects with bronchodilator reversibility <10% (PR, comprising 84% of the total population) corresponded to the ERS definition of COPD [18]. Results in the PR population were similar to the ITT population.

Withdrawals from clinical trials of COPD present a considerable problem in analysis and interpretation [24]. A significantly higher withdrawal rate on placebo/usual therapy (due to lack of potential benefits, thereby leaving a subset who may have less severe disease) may bias the results against the treatment. This imbalance between the treatment arms, with placebo/usual therapy results being artificially improved, would therefore reduce apparent treatment benefits. The meta-analysis showed a highly significant difference in the rate of early withdrawal from clinical studies between the treatment groups. Patients receiving salmeterol were up to 33% less likely to withdraw from studies than those receiving placebo/usual therapy (p < 0.0001). Furthermore, despite the greater withdrawal rate in the control group that may reduce apparent differences between treatments, highly significant treatment benefits with salmeterol were still detected. This suggests that salmeterol provided treatment benefits recognised and valued by patients that outweighed any potential side effects. Indeed, a recent meta-analysis has shown a good safety profile for salmeterol [25], supporting this hypothesis.

Lung function improved by 3.2%, 3.0% and 3.0% at 3, 6 and 12 months, respectively, suggesting that there was no tolerance, or decrease of efficacy, over time for up to 1 year of therapy. Some studies [26,27] have suggested that response may decrease with continued use whereas other studies have failed to find evidence of such tolerance [9,12]. The current meta analysis supports the latter studies although the reality and clinical relevance of any decrease continue to be an important focus of attention in COPD therapy.

Exacerbations are common in COPD and may have serious consequences [2,28,29]. Recovery from exacerbations may take more than a month in around a quarter of patients, or may even be incomplete [30]. A high frequency of exacerbations is associated with a more rapid decline in lung function [31], increased risk of hospitalisation [32] and reduced survival [33], with nearly half of patients hospitalised for a COPD exacerbation dying within 3 years [33]. A regular treatment that reduces the frequency and severity of exacerbations could, therefore, have an impact on both morbidity and survival. The current meta-analysis showed that salmeterol significantly (p < 0.0001) reduced the risk of experiencing a moderate or severe exacerbation by up to 28% compared with usual therapy. In addition to the potential patient benefits, this should also reduce medical resource utilization.

COPD is characterised by progressive decline in lung function of around 60–70 ml per year [34,35]. During an exacerbation, lung function falls by an average of 24 ml [30], while a short-term increase of 90 ml in patients with emphysema is sufficient to improve dyspnoea and exercise performance [36]. Consequently, the highly significant improvement in lung function of 73–86 ml observed with salmeterol compared with placebo/usual therapy is likely to be beneficial and clinically meaningful. Importantly, the measurements were made shortly before the next dose of study medication, representing the lowest value in the 12-hour dosing period, so that lung function at other time points could be expected to show a greater treatment difference. The difference between salmeterol and placebo/usual therapy was apparent from the earliest assessment point (3 months) and maintained at 6 months and 12 months, indicating that there was no decline in efficacy over this period.

There is increasing recognition that patient-centred outcomes, such as health status, are important in assessing the efficacy of medical interventions for COPD [37]. There was a consistent, statistically significant and clinically detectable improvement in health status in more patients treated with salmeterol than with placebo/usual therapy. A clinically meaningful change with the SGRQ (change in total score of 4 units) corresponds to patients, for example, 'no longer having to walk more slowly than other people, no longer being breathless on getting washed and dressed or on bending over' [38]. These changes are generally more relevant to patients than spirometric changes, although they are likely to reflect changes in the latter. The true extent of this benefit as a clinically relevant difference in health status has also been assessed and found to be greater with salmeterol.

The findings of the two populations analysed (Intent to Treat and Poorly Reversible) were consistent, indicating that the degree of reversibility to albuterol has little impact on response to salmeterol therapy, as reported previously [39]. There was some evidence of greater improvements in lung function with salmeterol treatment in those with better lung function at baseline, but all patients showed similar benefits in terms of exacerbation rate and health status. These findings suggest that salmeterol treatment is likely to benefit a wide spectrum of patients with COPD to a similar degree.

Recently, Salpeter et al. published the results of another meta-analysis of efficacy of various treatments in COPD [40]. Some of their conclusions appear initially to differ from ours, namely, that β2-agonists were associated with more respiratory deaths, and led to no difference in severe exacerbations, compared with placebo. The apparent differences are likely to reflect differences in aim, methodology and timing of the analyses. We analysed data only from studies of salmeterol, whereas Salpeter's methodology allowed consideration of any β2-agonist. In that analysis, most respiratory deaths – 60% of the weighting – were from a single trial of formoterol, its erratum, and accompanying unpublished data [40]. Similarly, 98% of the weighting for severe exacerbations was from a single study not included in our analysis. Thus, these results and ours presented here are not mutually contradictory.

It should be remembered that the underlying pathologies and concomitant medications in COPD and asthma are quite different. Nevertheless, recent major studies in asthma add to the body of knowledge about β2-agonists. In the SMART study, conducted in asthma patients, there was no significant difference between salmeterol and placebo in the primary combined endpoint of respiratory-related deaths or life-threatening experiences [41]. Respiratory-related deaths, and asthma-related deaths, were slightly but significantly more frequent with salmeterol, but these differences were not apparent after a 6-month follow-up period. Similarly, a meta-analysis conducted in asthma patients (in which SMART data contributed 80% of the weighted data) identified a greater risk of life-threatening asthma attacks with β2-agonists than placebo (6-month risk difference 0.12%; 95% CI 0.01–0.3%) [42]. Comparisons with the current data however cannot be drawn.

COPD is a multi-component disease, associated with inflammation, airway obstruction, mucociliary dysfunction and structural changes in the lung. Consequently, it is logical to assume that interventions addressing these different components will be more effective than treatments having more limited scope. In addition to effects on bronchodilation, salmeterol may have other effects, including promotion of mucociliary clearance, protection against bacterial-mediated epithelial damage and anti-neutrophil effects [43,44]. Whether these additional effects play a role in the overall benefit of salmeterol therapy has yet to be determined. The analysis showed consistently greater efficacy of salmeterol than with placebo when added to usual therapy, which included inhaled and oral corticosteroids, anticholinergic agents, methylxanthines and mucolytics. Importantly, there were no apparent trends in relative efficacy for studies with and without these medications, despite the expectation that a "usual therapy" comparator group may reduce treatment differences. This suggests that these other interventions target different components to salmeterol in the underlying pathophysiology of the disease. However, insufficient detail was collected in the studies to allow this to be examined specifically.

Since the meta-analysis was performed, other completed, published studies have been identified which could have been included in the analysis. In a small population of patients with moderate COPD, salmeterol (n = 6) over 52 weeks reduced exacerbations and lung function when compared to placebo but was less beneficial than a combination of salmeterol and fluticasone propionate [45]. In addition, results from two studies comparing salmeterol with the long acting anticholinergic tiotropium over 26 weeks have been published [46]. It was felt that inclusion of these studies would not alter the conclusion of the study due to the small patient numbers and in addition, we did not have the individual patient data for the analysis.

## Conclusion

In conclusion, this meta-analysis of nine large randomized clinical trials involving over 3500 patients in 34 countries has shown a consistent and highly statistically significant reduction in withdrawal rate from studies, reduction in exacerbation rate, improvement in lung function and improvement in health status with salmeterol compared with placebo/usual therapy, with no evidence of tachyphylaxis to bronchodilation over one year. The impact on a broad range of outcome measures suggests benefits from interventions for COPD that can modify more than one aspect of this multi-component disease.

## Competing interests

RS has no competing interests. PW and MW are employees of GSK who sponsored this meta analysis.

## Authors' contributions

RS was involved in the concept and planning of the meta analysis and in writing and editing the manuscript; PW and MW were involved in the writing and editing of the manuscript and responsible for the searches of the databases and the statistical analysis. All authors read and approved the final manuscript.
